# Scalable on-chip diffractive speckle spectrometer with high spectral channel density

**DOI:** 10.1038/s41377-025-01797-y

**Published:** 2025-03-20

**Authors:** Zimeng Zhang, Shumin Xiao, Qinghai Song, Ke Xu

**Affiliations:** 1 Guangdong Provincial Key Laboratory of Semiconductor Optoelectronic Materials and Intelligent Photonic Systems, Harbin Institute of Technology, Shenzhen, China; 2 Department of Integrated Circuits, Harbin Institute of Technology, Shenzhen, China

**Keywords:** Silicon photonics, Integrated optics, Metamaterials

## Abstract

The chip-scale integrated spectrometers are opening new avenues for a much wider range of applications than their conventional benchtop counterparts. While spectral reconstruction should be in command of both spectral resolution and bandwidth, a large number of spectral channels is among the key goals of the spectrometer design. However, the chip footprint eventually limits the spectral channel capacities of well-established spectral-to-spatial mapping structures like dispersive elements, filter arrays, random media, and so on. Here we suggest an alternative scheme by encoding the spectral information using on-chip diffractive metasurfaces. The in-plane metasurface is capable of producing intensity speckles to resolve the spectra. The spectral richness is greatly increased by scaling the architecture via three layers of cascaded metasurfaces. The readout of speckles is realized by two-dimensional imaging of the grating-diffracted pattern, enabling a large matrix for spectrum reconstruction. The spectrometer has a resolution of 70 pm over a bandwidth of 100 nm. Up to 1400 spectral channels were obtained within a compact chip area of only 150 μm × 950 μm. The on-chip diffractive spectrometer has a benchmark channel density of up to 10021 ch/mm^2^, which compares favorably against other state-of-art waveguide structures.

## Introduction

The needs for developing miniaturized spectrometer are growing fast due to a wide span of emerging applications in health care, agriculture, consumer electronics and so on^1–6^. Compared with conventional benchtop spectrometers, chip-scale spectrometers offer unique advantages in terms of size, weight, and power consumption (SWaP). By eliminating the bulk optics and moving parts that rely on precise alignment, the integrated spectrometers are more suitable for spectra measurement outside the laboratories. Making use of the proven integration platform of silicon microelectronics, silicon photonic integrated circuits offer the advantages of high reliability and easy scalability to large-volume and low-cost manufacturing^7–10^. The recent progresses on co-packaging unveil the potential opportunities for integration of both photonics and electronics in a compact form factor^11–13^.

Different types of miniaturized spectrometers have been realized on-chip while it remains a challenge to address the spectral channel density problem^14–17^. Since high spectral resolution is always desired in parallel with a broad operation bandwidth, the spectrometers should offer a large number of spectral channels which is normally defined as bandwidth/resolution. Several state-of-art spectrometer designs offer a considerable spectral capacity while they are associated with the issues of large footprints or data acquisition speed^18–20^. Specifically, dispersive components can be easily implemented by various structures, but they need substantial chip area to accumulate the optical path differences^21–27^. As an alternative, narrowband filtering could be constructed by an array of filters or a single tunable filter^28–36^. The available spectral channels are proportional to the number of filters or the tunable states which are eventually constrained by the chip area or response time. Similar issues are associated with the Fourier transform spectrometers as well^37–44^.

In addition to these carefully designed and controlled systems, speckle spectrometers based on disordered random media have been explored for spectra reconstruction as well. These structures well address the trade-off between the footprint and the spectral resolution^45–54^. However, the constraints on the available spectral channels remain challenges for broadband operation. For example, a speckle spectrometer using a random scattering structure can achieve a resolution of 0.75 nm, occupying an extremely compact chip area of only 25 μm × 50 μm. But, the operation bandwidth is 25 nm due to a limited number of spectral channels^45^. More recently, a two-dimensional (2D) photonic microring lattice was demonstrated with improved performance of 15 pm resolution over 40 nm bandwidth, offering up to 2666 spectral channels^20^. But the device structure occupies a chip area of 1 mm × 1 mm, which renders a limited channel density defined as channel-to-footprint ratio (CFR).

In this work, we proposed an integrated speckle spectrometer based on layered disordered metasurfaces. The in-plane optical guiding waves propagate through the metasurfaces where the diffraction behaviors are governed by the disordered meta-atoms with random structures. Speckle patterns can be created at the output plane of the metasurface and read by 2D imaging via a multimode grating coupler. The structure is scalable for multi-layers of metasurfaces to further increase the spectral channel capacity. The spectrometer based on three layers of metasurfaces can operate over 100 nm bandwidth with 70-pm resolution, and the footprint is only 150 μm × 950 μm. It indicates a benchmark large spectral channel density of 10021 ch/mm^2^. The foundry-fabricated spectrometer was fully compatible with the standard silicon photonic fabrication process. The proposed speckle spectrometer based on diffractive metasurfaces achieves a high spectral density which is promising to develop ultra-compact and high-performance spectrometers.

## Results

### Principle and design

**Fig. 1:**
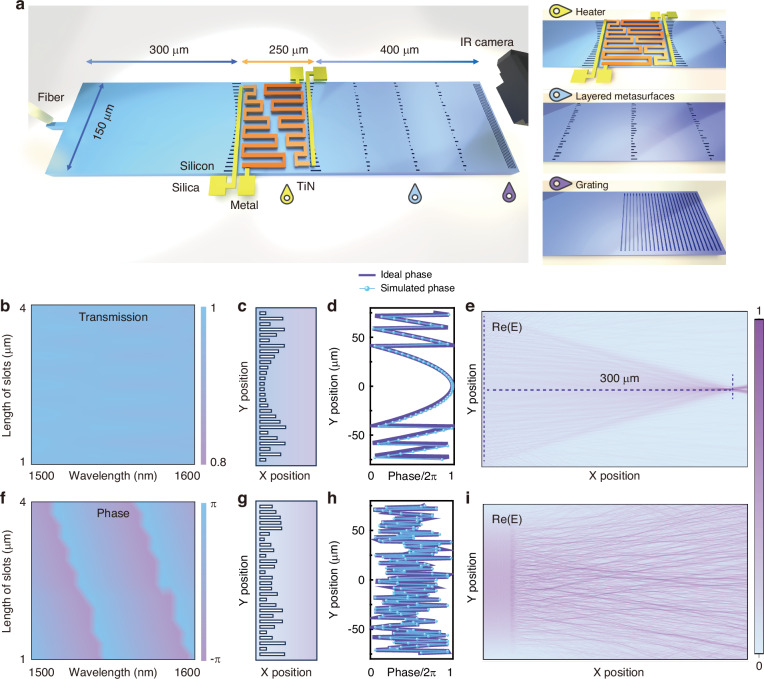
Structural schematic illustration and metasurface design. **a** The schematic diagram of the proposed spectrometer. **b** The simulated transmittance of the slot array. **c** The schematic diagram of the collimation metalens. **d** The phase profiles of the collimation lens. **e** The simulated optical field distribution of the lightwave collimation. **f** The simulated phase profile of the slot array. **g** The schematic diagram of the disordered metasurface. **h** The phase profiles of the disordered metasurface. **i** The simulated optical field distribution of the wave propagation through metasurface

The proposed spectrometer is designed on silicon-on-insulator (SOI) with 220 nm top silicon and 2 μm buried oxide. It consists of an input single-mode waveguide, a pair of collimated metalenses, three layers of diffractive metasurfaces, and a multimode output grating coupler. The schematic diagram of the device is shown in Fig. 1a where the insets zoom up the thermo-optic heater, the cascaded metasurfaces, and the output grating, respectively. The single-mode waveguide followed by a slab region was used as a diffused light source at the input. The diffracted light was collimated and focused by a pair of metalenses which were constructed via 1D array of etched waveguide slots, called phase gradient transmit array^55–58^. The metalenses have a focal length of 300 μm by proper design of the phase gradient. To avoid wavefront distortion, the first collimation metalens is used to create a plane wave for refractive index modulation via thermos-optic effects. The second lens is used for focusing the steered optical bean to different locations of the metasurface. Such a beam steering process can be achieved by thermo-optic modulation using the on-chip heaters^59^. The planar waves propagate through three layers of metasurfaces which were 100 μm away from the second metalens. In contrast to the phase gradient metalens, the metasurfaces modulated the planar waves with disordered phase distributions. According to the Huygens-Fresnel principle, the phase modulations from the meta-atoms can be converted to intensity modulations at the output plane. The spectral information can then be encoded to the speckle patterns at the output. A multimode grating coupler imaged the pattern on the camera followed by signal processing. According to the theory of the computational spectrometer, the input unknown spectrum was denoted as *S*(*λ*), and the matrix *I* represented the measured output speckles (fingerprints). If the transmission matrix $\text{T}$(*λ*) of the spectrometer was known by calibration measurements, the unknown spectrum can be resolved via ${\text{I}}_{\text{M}\times 1}={\text{T}}_{\text{M}\times \text{N}}\times {\text{S}}_{\text{N}\times 1}$, where M is the number of detection channels, and *N* is the number of spectral channels.

The basic concept of the metasurfaces is the phase modulation of the incident waves. The meta-atoms are created by etching a waveguide slot on the light propagation path. First of all, the meta-atoms should be lossless within the operation spectral range. Here, the width of each slot is chosen to be 180 nm to ensure a high transmittance, as shown in Fig. 1b where the numerically calculated efficiencies under different slot lengths and wavelengths are almost above 0.9. By controlling the slot length, the abrupt phase change of 0–2π can be introduced to the light field as seen by the phase diagram in Fig. 1f. This essentially makes it possible to manipulate the lightwave propagation. Due to the subwavelength scale of the etched slot, the phase response of the structure is wavelength-dependent, which is favorable for a reconstructive spectrometer. The structure of the collimation metalens is depicted by Fig. 1c. Its required phase mask and the structure-created phase profile are quite consistent as shown in Fig. 1d. The simulated optical field distribution in Fig. 1e reveals a 300 μm focal length, which matches with the design. The metasurface with randomly distributed meta-atoms and its corresponding phase mask are shown in Fig. 1g, h, respectively. Then, the optical field distributions of the metasurface can be found in Fig. 1i where a speckle pattern is obtained at the output plane. Thus, by using the assembly of subwavelength meta-atoms, the metasurfaces can be capable of simultaneously commanding both spatial and spectral features.

**Fig. 2:**
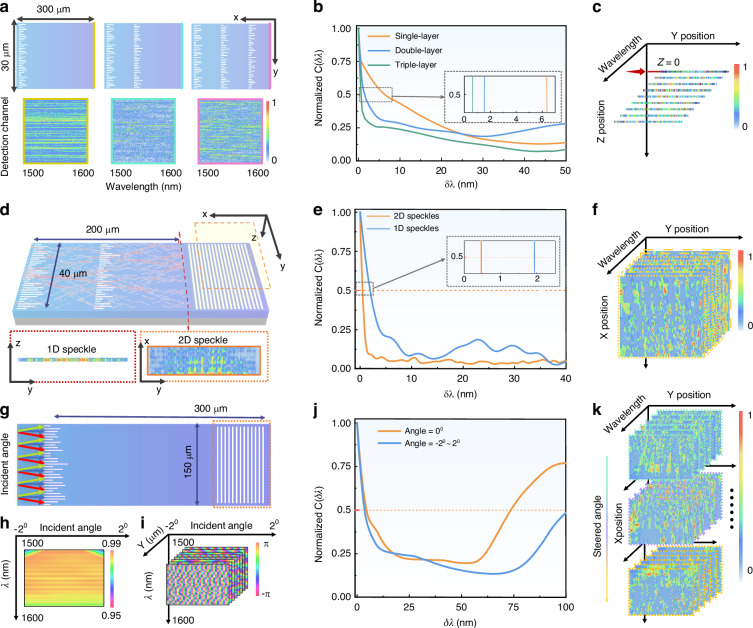
Performance evaluation of the scalable spectrometer. **a** Schematic diagrams and the speckle patterns of a multi-layer disordered metasurface. **b** The spectral correlation functions of different layers of metasurfaces. **c** The 1D speckle stack is directly sampled from the output plane in the slab waveguide. **d** A dual-layer disordered metasurface structure with a 1D speckle sampled in the output plane in the slab waveguide and the 2D speckle sampled from the grating diffraction output. **e** The spectral correlation functions of waveguide sampling and grating sampling scenarios. **f** The data cube obtained from 2D speckle. **g** The illustration of the wave vector steering approach. **h** Simulated transmission efficiencies with different steering angles. **i** The distribution of the wavefront phases with different steering angles. **j** A comparison of the spectral correlation function under fixed wave vector and wave vector steering conditions. **k** The scaled multiple data cubes obtained by combing the grating sampling and beam steering approaches

The proposed layered metasurfaces have great scalability to obtain a huge number of spectral channels without significantly increasing the footprint. First of all, multiple layers of metasurfaces introduce much more complicated wave propagation behaviors which eventually increase the effective interference path lengths. This phenomenon will be reflected by the speckle patterns containing more fine features of the spectra (i.e., spectral channels). This can be seen from Fig. 2a where the simulated intensity distribution at the output plane of three-layer metasurfaces exhibits more significant wavelength dependence than single-layer and dual-layer structures. Here, the 1D intensity profiles at different slices of the waveguide cross sections were obtained by field monitors implemented in the 3D simulation model. This advantage can be partially revealed by the correlation function which is normally used for quantitatively investigation of spectral resolution. Given the spatial intensity distributions obtained at different wavelengths, the correlation function can be calculated by:

1 $$C\left(\Delta \lambda \right)={\left\langle \frac{{\left\langle I\left(\lambda ,x\right)I\left(\lambda +\Delta \lambda ,x\right)\right\rangle }_{\lambda }}{{\left\langle I\left(\lambda ,x\right)\right\rangle }_{\lambda }{\left\langle I\left(\lambda +\Delta \lambda ,x\right)\right\rangle }_{\lambda }}-1\right\rangle }_{x}$$

where $I\left(\lambda ,x\right)$ is the recorded intensity at position *x* for wavelength *λ*. The ${\left\langle \cdots \right\rangle }_{\lambda }$ and ${\left\langle \cdots \right\rangle }_{x}$ correspond to the average over wavelengths and spatial channels respectively. In Fig. 2b, the spectral correlation curves were calculated via the simulation results. The half-width-half-maximum of $C\left(\Delta \lambda \right)$ implies the required minimum wavelength shift to reduce the spectral correlation by half, which is an indicator of the capability for distinguishing the narrowband spectral components close to each other. The inset shows the zoom-up features of the correlation curves which indicate a much narrower correlation width obtained by the three-layer structure. For a reconstructive spectrometer, it implies better spectral resolution or more channels available within the spectral range from 1500 nm to 1600 nm. By sweeping the input wavelength in the calibration process, a stack of 1D speckle patterns can be obtained at the output plane and used for spectral reconstruction, as shown by Fig. 1c. The richness of the spectral information contained in these patterns essentially reflects the performance of a speckle spectrometer.

Rather than spatially sampling the 1D speckle pattern via waveguide array, here a multimode grating was used to expand the 1D intensity distribution to 2D speckle pattern by diffracting the in-plane waves to the imaging camera in free space. Here, we consider dual-layer metasurfaces to verify the advantage of the 2D speckle imaging scheme. The schematic diagram of the structure is referred to Fig. 2d. The inset figures represent the simulated 1D optical field at the metasurface output plane and the diffracted 2D optical field, respectively. The correlation functions of both 1D and the 2D speckle patterns were calculated and shown in Fig. 2e. By looking into the zoom-up curves, the 2D speckle exhibits a narrower correlation width than 1D speckle, which reflects a better resolution as expected. In Supplementary Note 1, this advantage can be validated by detail comparative performance analysis of a dual-layer metasurfaces spectrometer via 1D speckles obtained by waveguide array and 2D speckles obtained by multimode grating. By 2D speckle imaging approach proposed in this work, the data stacks obtained by cascaded metasurfaces can be scaled to a large data cube shown in Fig. 2f. This method compares favorably with the conventional spatial sampling using 1D waveguide array in which the number of channels is constrained by the chip area. It also eliminates the risk of losing spectral information due to the required physical spacing between adjacent channels.

Within a specified wavelength range, the spectral resolution is relevant to the available wavelength channel capacity of the speckle. Inspired by the angular sensitivity of nonlocal metasurfaces in free space^60–62^, the spectral channel capacity can be further scaled by detuning the wavefront via a specifically designed on-chip heater. By injection of electrical currents to the heater strips as shown in Fig. 1a, the local refractive index can be modified with a desired distribution via thermos-optic effect. Such an index profile introduces phase gradient to the wavefront, which eventually steers the propagating wave vector as indicated by Fig. 2g. The heater design and the beam steering performance are described in detail as referred to Supplementary Note 2. The light propagation with a small steered angle within ±2° has negligible impact on the transmission efficiency which was validated by the results in Fig. 2h. The angle-steered scheme introduces an additional degree of freedom to control the wave propagation. Indicated by Fig. 2i, the phase changes induced by metasurface can cover full 2π range under different steered angles as well. This bonus is credit to the changes of wave propagation behaviors under different wave vectors. By the combination of all these features, the spectral capacity can be eventually scaled to a tremendous level as indicated by the multiple data cubes shown in Fig. 2k. The correlation functions of the speckle patterns with and without the steering angle are calculated, as shown in Fig. 2j. The result reveals that the control of the deflection angle expands the working bandwidth of the spectrometer theoretically. These scalabilities of the proposed structure enable the spectrometer to operate adaptively according to different applications.

### Calibration of the spectrometer

**Fig. 3:**
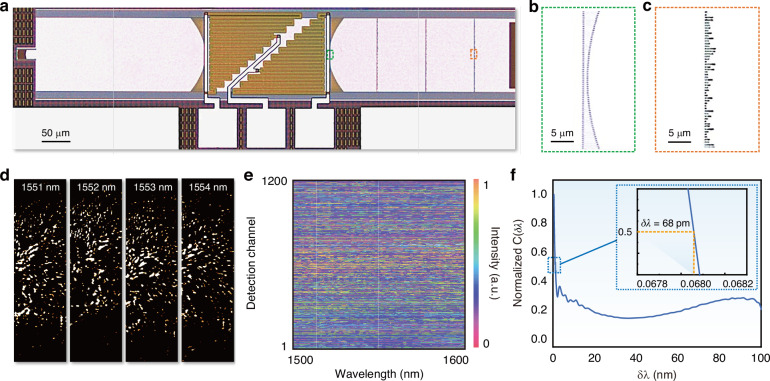
Experimental characterization of the fabricated spectrometer. **a** The optical microscope image of the device. The zoom-up images of: **b** the metalens and **c** the disordered metasurface. **d** The measured output speckle patterns at input wavelengths of 1551 nm, 1552 nm, 1553 nm, and 1554 nm, respectively. **e** The merged speckle matrix consists of measured intensities of all the wavelengths in each channel. **f** The normalized spectral correlation function of the fabricated spectrometer

The proposed spectrometer was fabricated via multi-project wafer shuttle run offered by a commercial silicon photonic foundry. The detail fabrication process can be referred to as Methods. The microscope image of the whole device is shown in Fig. 3a. It occupies a compact chip area of 150 μm × 950 μm. Three electrodes with common ground configuration were used on a pair of heaters for wavefront phase modulation. The zoom-up features of the phase gradient metalens and disordered metasurface are shown by the microscope images in Fig. 3b, c, respectively.

Prior to the reconstruction of an arbitrary spectrum, the device was first calibrated by capturing the wavelength-dependent speckle patterns across the whole operation spectral range. We recorded the spectral-to-spatial mapping to create the required data cubes for the following spectral reconstructions. The sweeping laser output was coupled to the chip via a single-mode grating coupler with efficiency of −5.5 dB/facet. The wavelength stepping size is chosen to be 5 pm over the whole range from 1480 nm to 1640 nm which is limited by the available laser source. The output diffracted patterns from the multimode grating coupler were imaged by an InGaAs infrared camera. We select four sets of speckle patterns under illumination from 1551 nm to 1554 nm as shown in Fig. 3d, respectively. The difference among them implies significant wavelength dependencies among the measured speckle patterns (i.e. spectral fingerprints). The complete data of spectral-to-spatial mapping can then be obtained as shown in Fig. 3e. Here the multiple data cubes have been merged into a large matrix for easy data processing. It is worth noted that the conventional on-chip spatial sampling method via waveguide array inevitably losses spectral information due to the waveguide spacing between the neighboring channels. Here, the proposed imaging scheme well preserves all the spectral information. The digital imaging processing offers great versatility for data acquisition. The detail process of image processing can be found in Supplementary Note 3. Thanks to the scalability of the proposed spectrometer, it comes up with thousands of available data channels for spectral reconstruction. This is a considerably large capacity compared with other on-chip counterparts, since the chip area constraints would not allow for so many detectors. Given the measured spectral-to-spatial matrix merged from the data cubes, the correlation function was calculated and plotted in Fig. 3f. As seen from the inset zoom-up figure, the minimum decorrelation wavelength of the transfer matrix was estimated to be *δλ* = 68 pm.

### Spectral reconstruction

Generally the spectral reconstruction can be interpreted by finding the input unknown spectrum *S*(*λ*) by minimizing ${{\rm{||TS}}-{\rm{I||}}}^{2}$ where ${{||}\cdots {||}}^{2}$ indicates taking the norm. The *I* represent the measured speckles (fingerprints), and the transmission matrix $\text{T}$(*λ*) of the spectrometer has been obtained via the calibration process. While such transfer matrix method is normally vulnerable to experimental noise induced by equipment, temperature drift, environmental vibrations and so forth, the compressed sensing approach based on CVX was employed in this work to enhance the system robustness. Additionally, to achieve accurate reconstruction for both sparse and continuous spectra, we incorporate both *l*1 and *l*2 regularization terms^34^ as expressed in the following equation:

2 $${\min }_{\text{S}}\left(||{\text{TS}}{-}{\text{I}}||^{2}+{{\alpha }}_{1}||{\text{S}}||_{2}^{2}+{{\alpha }}_{2}||{\rm{S}}||_{1}\right),{\text{subject}}\,{\text{to}}\,0\, <\, {\text{S}} \,<\, 1$$

where α_1_ and α_2_ represents the weight coefficient of *l*1 and *l*2 regularization respectively. It is important to note that the value of ${a}_{1}/{a}_{2}$ is relevant to the spectra properties. While a large value of ${a}_{1}/{a}_{2}$ is more efficient for reconstruction of continuous spectra, smaller ${a}_{2}/{a}_{1}$ is more appropriate for reconstruction of the discrete spectra. The adaptive configuration of these weight coefficients can be possibly achieved by the generalized cross-validation statistic and deep learning models^63^.

**Fig. 4:**
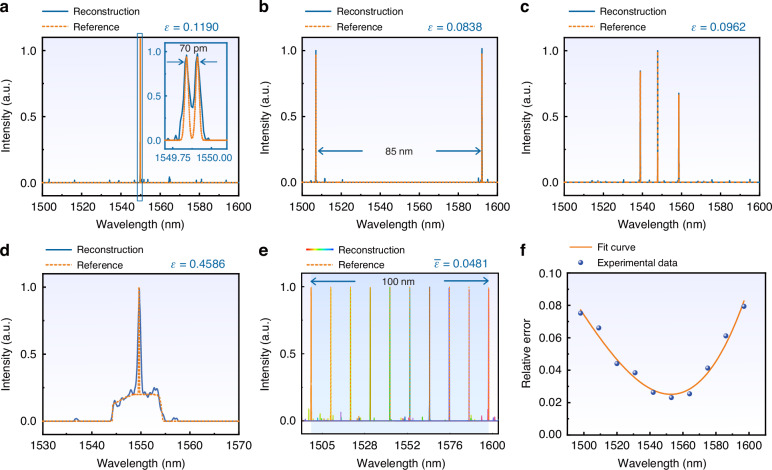
Experimental results of arbitrary spectrum reconstructions. **a** The reconstructed spectrum of dual spectral lines with a spacing of 70 pm. Inset: zoom-up feature of the curves. **b** The reconstructed spectrum of a double-peak with a spacing of 85 nm. **c** The reconstruction of three peaks with different intensities. **d** The reconstructed spectral of the mixed spectrum consists of a narrowband spectral line on a broadband background. **e** The reconstructed spectra of a series of spectral lines within a 100 nm wavelength range (1498–1598 nm). **f** The relative reconstruction error *ε* at different wavelengths

To validate the capabilities of arbitrary spectra reconstruction, various types of spectra were reconstructed by the proposed spectrometer for a proof-of-concept demonstration. The reconstruction results of the proposed spectrometer (solid line) are compared with the measurement data from a commercial optical spectrum analyzer (OSA, YOKOGAWA AQ6370C) (dashed lines). Firstly, the resolution of the spectrometer was evaluated by dual-peak measurement where two closely spaced spectral lines were generated by two sets of narrow-linewidth lasers. The spectra obtained by the commercial OSA and the proposed spectrometer are shown in Fig. 4a. As seen from the inset, spectral lines with a minimum separation of 70 pm can be differentiated by the spectrometer which is consistent with the theoretical resolution shown in Fig. 3f. The dual-peak spectrum measured by OSA with 20 pm resolution is also plotted in the inset for reference. The spectrometer is also capable of reconstructing two spectral lines with a wide separation of 85 nm as illustrated in Fig. 4b. It is also worth considering the multiple wavelength peaks with different amplitudes. In Fig. 4c, three wavelengths with different spacing and amplitudes were successfully reconstructed with perfect match with the spectra obtained by OSA. To demonstrate the capability of arbitrary spectrum reconstruction, a more complex spectrum consisted of a narrowband Gaussian peak with a flat-top broadband background was measured as seen from Fig. 4d. The spectrum was created by a broadband light source and a tunable laser (Santec TSL-710). While discrepancies can be observed, the reconstructed spectrum is almost consistent with the original spectrum. It well validates the universal operation capability of the proposed metasurface spectrometer. To further verify the operation bandwidth of the spectrometer, a series of spectral lines were generated across the 1500 nm to 1600 nm range using a tunable laser and were reconstructed individually. As shown in Fig. 4e, all the spectral pieces were successfully reconstructed with good accuracy, which implies the broad optical bandwidth (>100 nm) of the spectrometer. To evaluate the accuracy of the spectra reconstruction, the relative reconstruction error can be quantified as follows:

3 $$\varepsilon =\frac{{\left[{\sum }_{i=1}^{M}{\left(S-{S}_{0}\right)}^{2}\right]}^{0.5}}{{\left[{\sum }_{i=1}^{M}{{S}_{0}}^{2}\right]}^{0.5}}$$

where *S* and *S*_0_ denote the recovered and the original spectrum, respectively. *M* is the number of wavelength channels. For broadband wavelength peak reconstruction in Fig. 4e, the calculated errors ε range from 0.0231 to 0.0736 as seen from Fig. 4f. The average error is estimated to be 0.0481, which indicates a high reconstruction accuracy. The relative reconstruction errors of all the scenarios mentioned above have been labeled in Fig. 4. The single peak over broadband background spectrum reconstruction has slightly larger error of 0.459 due to the complexity of the spectrum, which consists of both narrow and broad spectral components. It can be seen from these reconstructed spectra, the speckle spectrometer is more vulnerable to the background noise, as the speckle spectrometer normally has lower sensitivity for weak signals. This is because the speckle spectrometer intrinsically divides even a monochromatic light into many detectors instead of a single detector, which lowers the sensitivity. The absolute intensity of the reconstructive spectrum could be measured by calibration of the system insertion loss in a similar way to the commercial spectrometer.

## Discussion

**Fig. 5:**
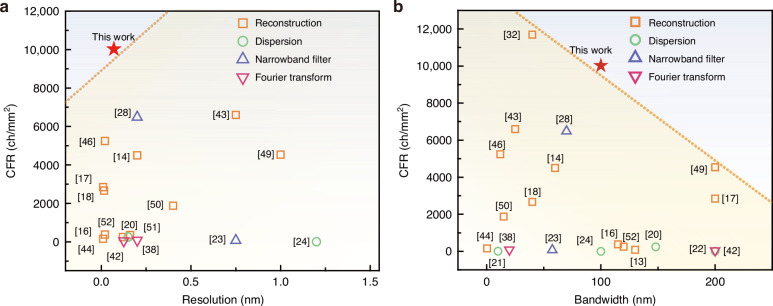
Summary of recent advances of the state-of-art spectrometers. The performance is compared in terms of CFR versus. **a** spectral resolution (*δλ*) and **b** optical bandwidth

For a speckle spectrometer, the capabilities of arbitrary spectrum measurement with high resolution and broad bandwidth are highly related to the available wavelength channels. It normally has a large channel capacity compared with conventional dispersive spectrometers and Fourier spectrometers. The on-chip integration of both speckle pattern generation and readout comes up with great challenges for a large number of channel counts. As mentioned, the readout of spectral-to-spatial mapping requires a densely packed waveguide and detector array to accommodate all the spectral channels, which significantly increases the footprint. The spatial sampling via waveguides inevitably losses spectral information due to the waveguide spacing as well. Here, we applied the multimode grating and imaging camera as an alternative, which offers the advantages of great flexibility in imaging processing and compactness. In Fig. 5a, we summarized the recent advances in state-of-art on-chip spectrometers and made a performance comparison. Even though the speckle spectrometers have greatly improved the spectral resolution due to their capabilities of creating more spectral channels, a trade-off exists between the resolution and the compactness. This can be reflected by the reduction of CFR with an improvement in spectral resolution. As indicated in the left upper corner of the figure, our proposed spectrometer has overcome this barrier with a benchmark high channel density.

In pursuit of high spectral resolution, the optical bandwidth is normally constrained. Intuitively, both features can be improved simultaneously by incorporating more spectral channels. However, the difficulty is associated with the required large chip area to do so. Thus, it makes sense to improve the spectral channel density for miniaturized and high-performance spectrometers. As seen from Fig. 5b, most of the reported high-resolution speckle spectrometers have limited bandwidth. Extended operation bandwidth can be possibly achieved by increasing the CFR as implied by the trend on this curve. Our work compares favorably against the others in terms of CFR and goes well beyond the existing records. The proposed spectrometer has excellent scalability allowing for performance improvement via further increased CFR. A comprehensive performance summary of the recent advances in integrated spectrometers can be found in Supplementary Note 4.

In conclusion, previous roadblocks for compact, broadband, and high-resolution spectrometers can be possibly overcome by one of the promising designs proposed in this work. The spectrometer was fabricated via a commercial foundry via a standard silicon photonic process. It is fully compatible with the mature integration platform of silicon microelectronics, which offers the advantages of high reliability and easy scalability to large-volume and low-cost manufacturing. The layered meta-surface structure serves as a new type of speckle spectrometer and offers excellent scalability to further increase the CFR for better performance. The metasurface speckle spectrometers can also be integrated on various material platforms for extended wavelength ranges in visible light or mid-IR regimes.

## Materials and methods

### Device fabrication

The device was fabricated in a commercial silicon photonic foundry via a multi-project wafer shuttle run. The waveguide layer was defined by the 193 nm deep UV lithography. Inductively coupled plasma dry etching was used to deeply etch the grating coupler with 70 nm depth and to deeply etch the strip waveguide with 220 nm depth, respectively. Then the waveguides were cladded by thermal oxidation followed by planarization. The heaters were fabricated by deposition of 0.1 μm-thick TiN followed by contact via opening and bond pad formation. The minimum feature size of the spectrometer is 180 nm which can be well fabricated in this process.

### Optical measurements setup

The experimental setup for device characterization consists of a 1550 nm sweep laser (Santec TSL-710), a fiber-based polarization controller, a fiber-chip coupling stage, a benchtop power meter (MPM210), and an infrared camera (LUSTER-CB2000). The single-mode fibers are used to probe the I/O grating couplers. The polarization controller was used to excite the TE guiding mode in the waveguide. The transmission spectra are acquired by measuring the output powers with wavelength swept from 1480 to 1640 nm with a step size of 5 pm. The multimode grating output speckle is captured by the IR camera at the far field. The imaging plane consists of 1280 × 1024 pixels with a pixel size of 5 μm × 5 μm. For arbitrary spectrum reconstruction, the incident spectra were created by a broadband light source, a narrow-linewidth sweep laser, and a programmable filter (Viavi MTFX-C1).

## Supplementary information

Supplementary Information

## Data Availability

The data that support the findings of this study are available from the corresponding authors upon reasonable request.
