# Supplementary material for: Scalable on-chip diffractive speckle spectrometer with high spectral channel density
Source: Light Sci Appl. 2025 Mar 20;14:130. doi: 10.1038/s41377-025-01797-y (PMC11926203; doi:10.1038/s41377-025-01797-y)
Supplement: Supplementary file 1 — Supplementary Information [file 41377_2025_1797_MOESM1_ESM.docx]

**Supplementary Information for**

Scalable on-chip diffractive speckle spectrometer with high spectral channel density

Zimeng Zhang^1,2^, Shumin Xiao^1^, Qinghai Song^1,2*^, and Ke Xu^1,2*^.

1. Guangdong Provincial Key Laboratory of Semiconductor Optoelectronic Materials and Intelligent Photonic Systems, Harbin Institute of Technology, Shenzhen 518055, China
2. Department of Integrated Circuits, Harbin Institute of Technology, Shenzhen 518055, China

* Corresponding author: Qinghai Song ([qinghai.song@hit.edu.cn](mailto:qinghai.song@hit.edu.cn)), Ke Xu ([kxu@hit.edu.cn](mailto:kxu@hit.edu.cn)).

**Contents:**

Supplementary Note 1│Comparative analysis of the spectrometer via 1D and 2D speckle.

Supplementary Note 2│The heater design and beam steering performance.

Supplementary Note 3│Image processing.

Supplementary Note 4│Performance summary of state-of-art on-chip spectrometers.

**Supplementary Note 1│Comparative analysis of the spectrometer via 1D and 2D speckle.**


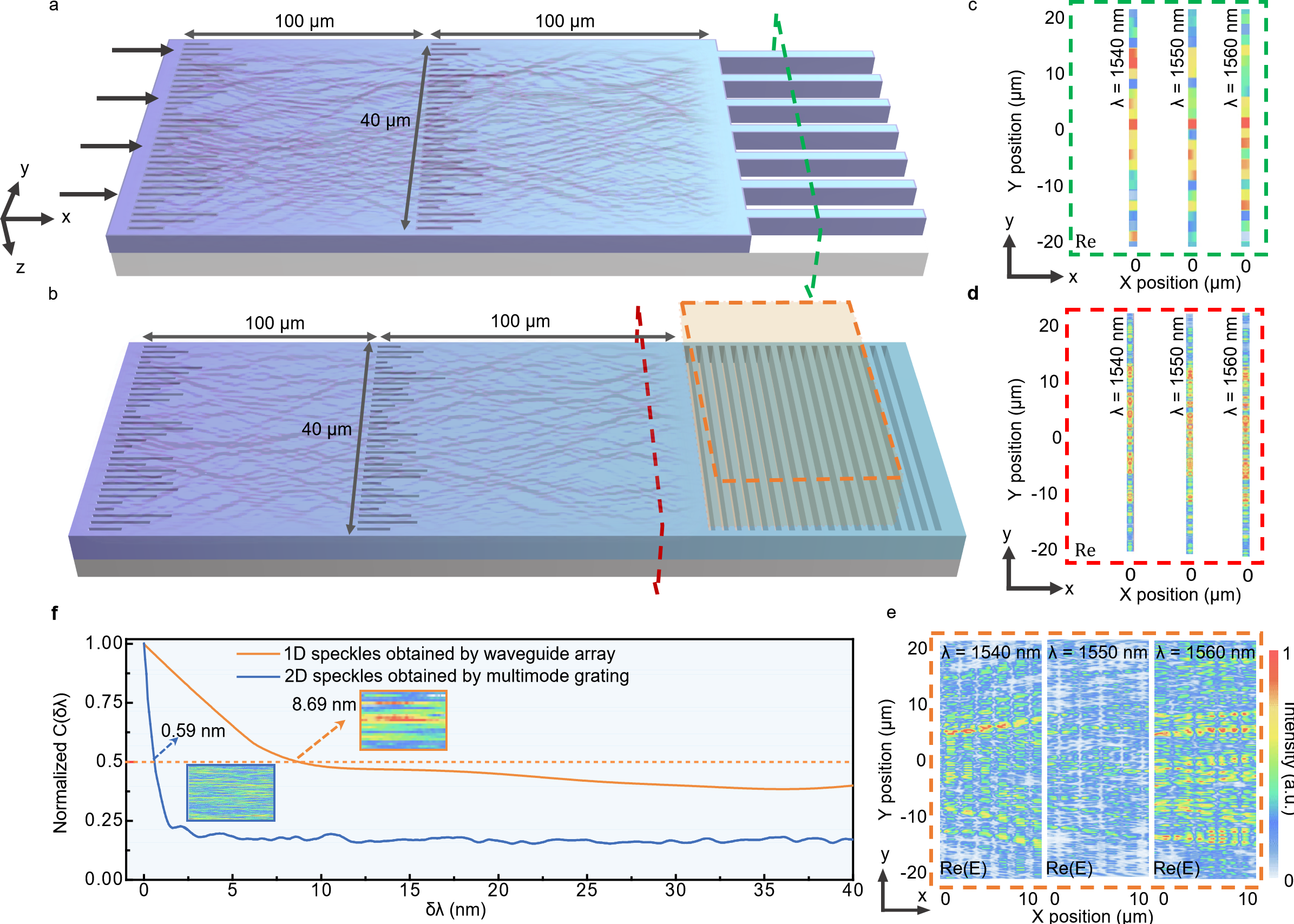


Fig. S1 | Comparative analysis of 1D speckle sampled by waveguide array and 2D speckle sampled by grating with imaging camera. The schematic diagram of a dual-layer metasufaces **a** sampled by single-mode waveguide arrays; **b** sampled by multimode grating with imaging camera. **c** The 1D speckles under-sampled by waveguide array. **d** The 1D slices (position x=0) of 2D speckle fully-sampled by multimode grating. **e** The scaled 2D speckles obtained by multimode grating with imaging camera. **f** The comparison of the spectral correlation function under 1D speckles under-sampled by waveguide array and 2D speckles obtained by multimode grating with imaging camera.

To validate the advantages of the 2D speckle sampled by multimode grating with imaging camera over 1D speckle sampled by waveguide array, a comprehensive analysis was performed. Here we consider a dual-layer metasurfaces for a proof-of-concept demonstration. The structures of the 1D sampling and 2D sampling devices are depicted by Fig. S1a and b, respectively. The metasurfaces have a width of 40 μm and the free-propagation distances are 100 μm. As seen in Fig. S1c, the discrete waveguide and detector array only record 1D intensity distributions in y-direction. Though wavelength dependence can be observed, these 1D speckles are under-sampled due to the waveguide gap between adjacent channels. It eventually loses a considerable amount of spectral information which limits the spectrometer performance. In contrast, the multimode grating well avoids this issue by recording continuous intensity distributions in y-direction. The slices (x=0) of speckles under different wavelengths are shown in Fig. S1d. The speckles obtained by multimode grating contain much more fine features which imply a better performance.

Another benefit of the proposed scheme is the capability to scale the speckles from 1D to 2D. The multimode grating diffracted the metasurfaces output and expanded the speckle in x-direction This essentially extends the speckle patterns from 1D to 2D. The infrared camera has a large number of pixels which is able to record a continuous pattern in far field. The scaled 2D speckles under different wavelengths can be seen in Fig. S1e. More pronounced wavelength dependence can be observed by examining the 2D patterns. This is primarily due to the more sophisticated mapping between spectrum and speckle via additional diffraction and interference process. To quantitatively compare the performance between 1D and 2D speckles spectrometers, the correlation analysis was performed as shown in Fig. S1f. The 2D speckle patterns allow for much narrower correlation width which implies a much better spectral resolution. The spectral resolution of the 2D speckle approach is estimated to be 0.59 nm while the 1D speckle approach has a resolution of only 8.69 nm.

**Supplementary Note 2│The heater design and beam steering performance**

In order to realize the on-chip beam steering, the targeted wavefront phase profile (Δ𝜑) induced by the free-form microheater can be determined by the following equation.

$$\begin{aligned} \varphi\left( y \right)=\frac{2\pi}{\lambda}n_{\mathrm{slab}, eff}\left( y+\frac{D_{\mathrm{lens}}}{2} \right)\frac{y_{h\mathrm{eater}}}{f}\#\left( s1 \right) \end{aligned}$$

where 𝜆 is the operation wavelength, 𝑛_slab, eff_ is the effective refractive index of the slab silicon waveguide (𝑛_eff_ = 2.86), 𝑓 is the designed focal length of the metalens, y_heater_ is the focus lateral (y-axis) offset distance after heating. According to Eq. S1, the wavefront phase should change linearly along the y-axis direction for beam steering. Therefore, the heater is designed to be a triangular-geometry with linear gradient as shown by the schematic diagram in Fig. S2a. The heater has a maximum length of 220 μm and a width of 160 μm. The length of the heater strip varies from 14 μm to 220 μm with 15 μm step difference. The cross-sectional schematic diagram of the designed heater structure is shown as Fig. S2b. The thermal conduction and thermo-optic effects were modeled by finite element method. The numerically simulated temperature and effective index distributions under 100 mW heating power on heater 1 are shown in Fig. S2c and S2d, respectively. A gradient in both temperature and refractive index profile can be observed which is almost consistent with the designed heater geometry. It indicates that arbitrary index profile can be potentially achieved by proper heater design. By applying heating power on heater 1 and heater 2, bi-directional wavefront steering can be achieved. This can be validated by the wavefront phases calculated in Fig. S2e. The detail simulation model of the beam steering device can be referred to our previous work (Laser Photonics Rev. 17, 2300330, 2023). Here, a pair of back-to-back metalenses are investigated in the simulations as seem from Fig. S2f. By tuning the heating power from 0 to 500 mW on heater 1 and heater 2 respectively, the focal point intensity peaks can be steered from output port 6 to port 1 and from port 6 to port 11, accordingly. This can be observed from the result shown in Fig. S2g. A more straightforward result is shown by the optical field distribution shown in Fig. S2h. By turn-on of heater 1 and heater 2, the optical beam has been steered downwards and upwards with 12.5 μm off-set in y-direction.


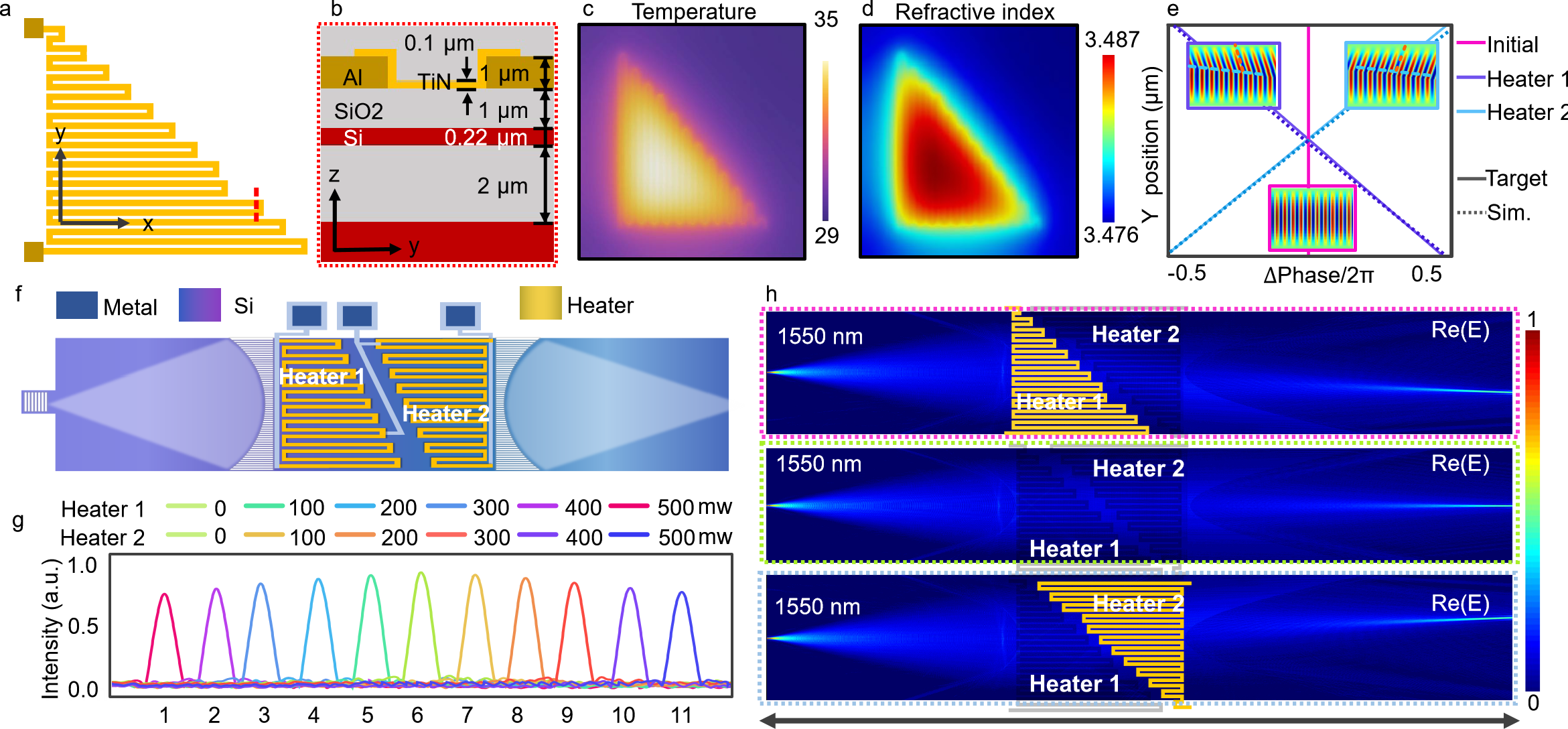


Figure S2| The design and simulated performance of the microheater. **a** The top-view and **b** cross-sectional schematic diagram of the designed heater structure. **c** The calculated temperature and d refractive index distribution of slab waveguide under 100 mW electrical power applied to the heater. **e** The phase profiles of the wavefronts under with and without thermo-optic tuning. **f** The schematic diagram of tunable metalens for beam steering. **g** The normalized intensities at the focal plane under different electrical powers applied to heater1 and heater2. **h** The simulated optical fields under different tuning schemes.

The performance of the on-chip beam steering has been experimentally characterized as shown in Fig S3. A testing structure for beam steering was fabricated on the same chip with the spectrometer. The microscope image of the test structure is shown in Fig. S3a. Eleven output waveguide with grating I/O were designed to measure the steered intensity peaks. Fig. S3b depicts the waveguide sampling structure at the output focal plane. The width of the sampling waveguide is chosen to be 2.3 μm for increasing the collection efficiency while the waveguide gap distance is 0.7 μm to avoid crosstalk (CT). In Fig. S3c, the intensity peak measured at the focal plane shifts from central port (O6) to O3 and O9 by applying 321 mW heating power on heater 1and heater 2, respectively. The experimental results agree well with the simulation prediction as shown in Fig. S3d. Slightly higher loss is observed in output channels on two sides, which is due to the wave vector mismatch between the steered wave and the output waveguide. This can be further improved by adjustment of the output waveguides with a tilt angle. The worst CT was measured to be -12 dB as shown in Fig. S3e. The insertion losses at 1550 nm are measured to be 3~4.8 dB for all the channels. For real-time operation of the spectrometer, the heater should be driven by square wave electrical signals. Here, we characterize the response speed of the wave vector steering process by applying a square wave electrical signal with frequency of 5 kHz and amplitude of 6 Vpp. The measured rise time and fall time are 42 μs and 37.8 μs, respectively. The frame rate of the camera is 134 fps which corresponds to 7.5 ms per frame. For eleven tuning states required for beam steering from -2° to 2°, the corresponding period of time for one tuning cycle can be estimated to be (42 μs + 37.8 μs + 7.5 ms)×11=83.4 ms. This is negligible compared with the following imaging processing (0.12 s) and spectrum reconstruction (0.43 s) for a fully sampled data matrix with size of 1200×20000. The proposed spectrometer can be flexibly configured by either turning on or turning off the heater according to different requirements. For both scenarios, fast operation with <1 s frame rate is achievable.


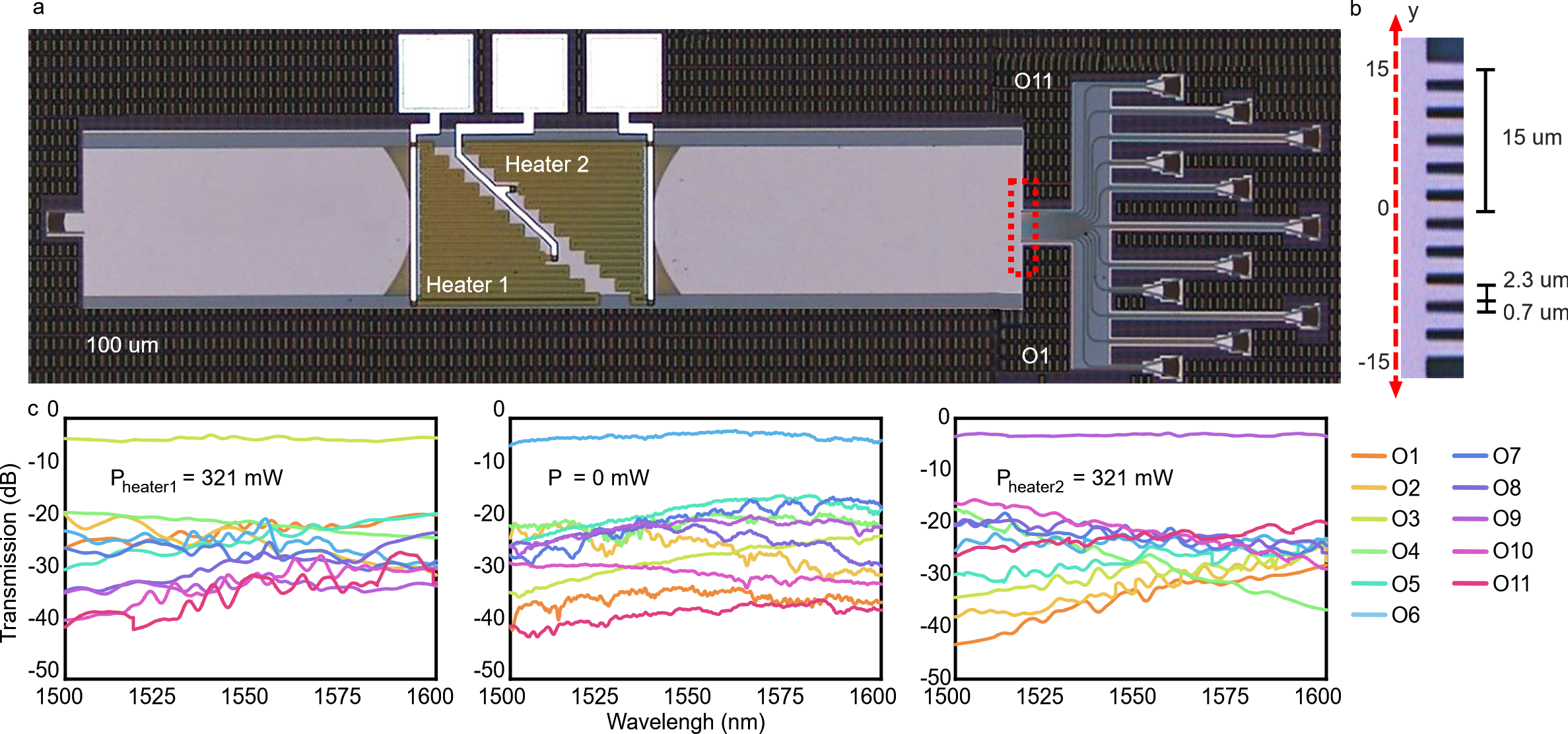


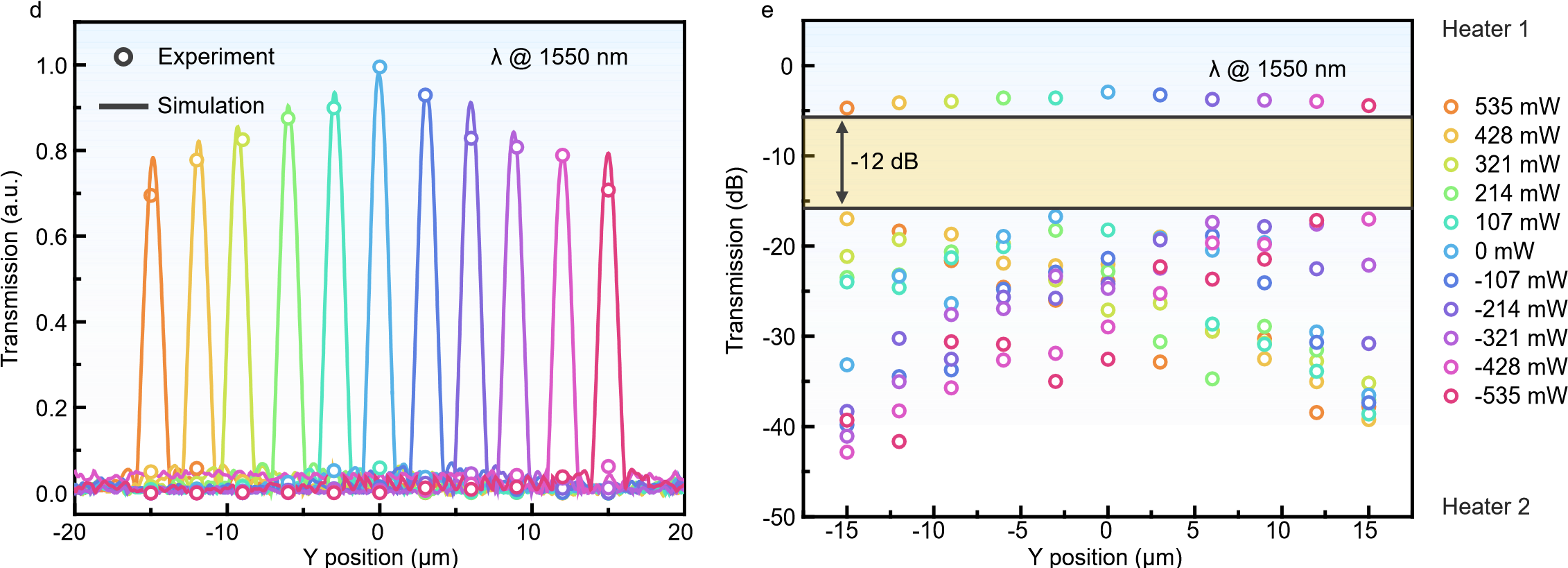


Figure S3| The design and simulation performance of the microheater structure. **a** The microscope image of the fabricated back-to-back meta-lens for beam steering characterization. **b** Zoom-up feature of the output waveguide array at the focal plane. **c** The measured transmission spectra of all the output ports from 1500 nm to 1600 nm by turn-off and turn-on of the heater. **d** The simulated and measured optical intensity peaks of each output port at the focal plane at 1550 nm. **e** The measured transmission of different ports at 1550 nm under different heating powers.

**Supplementary Note 3│Image processing.**


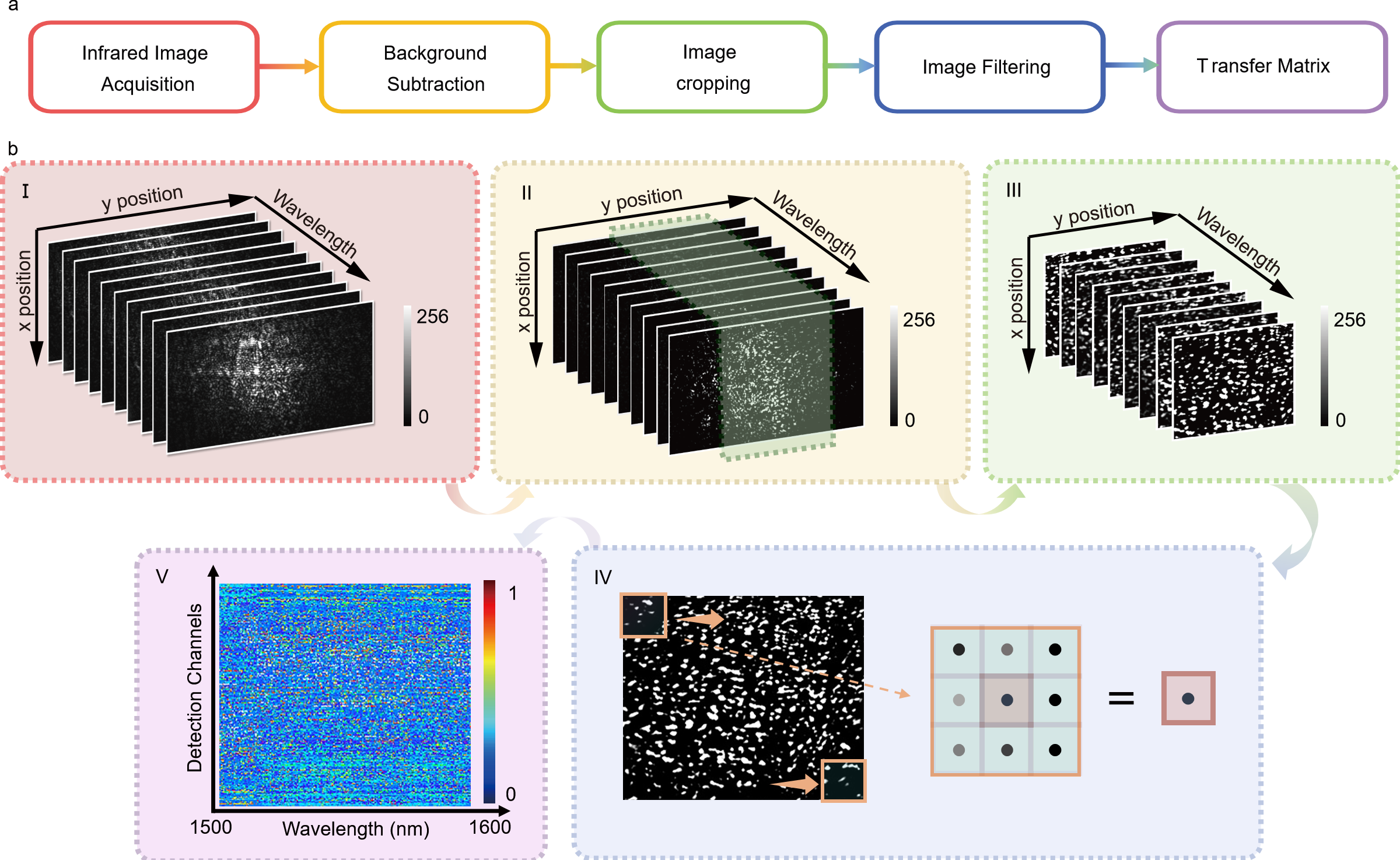


Figure S4| The imaging processing of the speckles. **a** Simplified flow of the image processing. **b** Schematic diagrams for each corresponding step in the flow.

In this experiment, an infrared camera was utilized to capture the 2D speckle patterns diffracted from the multimode grating output. Given the redundancy of information within the images, direct processing of the speckle patterns takes too much reconstruction time and suffers from noise problem. As shown in Fig. S4, a series of preprocessing steps were performed to retrieve the transfer matrix. The procedure can be described by the following steps:

I) **Infrared images acquisition**: Initially, the optical set-up was optimized to obtain the speckle images with high qualities. The images were converted to raw matrices for further processing.

II) **Background subtraction**: In order to extract the spectral features of the images, the digital technique of background subtraction has been applied in this step. The background images were first identified and extracted according to certain thresholds. They are subtracted from the speckle images obtained in step I). Then, the intensities of each image were normalized with 8 bits.

III) **Image cropping**: By employing edge detection algorithms, precise identification of speckle regions within images can be achieved, as depicted by the green dash line in Fig. S4b. Following identification, an image segmentation operation is performed to retain regions enriched with speckles (step III in Fig. S4b). This step can effectively eliminate most irrelevant background elements and interfering noise. This targeted segmentation not only enhances data processing accuracy but also boosts the efficiency and precision of the overall processing workflow.

IV) **Image filtering**: The presence of noise in images deteriorate the accuracy of the reconstruction, especially for spectra with both broadband and narrowband features. To enhance the reconstruction robustness against noise and preserve spectral information simultaneously, median filtering was employed here (step IV in Fig. S4b). As a non-linear filtering technique, this method is able to retain image details while effectively mitigating the impact from noise. The convolution kernel with an optimal window size of 13 × 13 has been used here for transmission matrix generation.

V) **Transfer matrix**: Finally, a set of 3D data cubes consists of spectral features can be obtained from step I-step IV. These 3D data cubes were merged into a 2D transmission matrix. This matrix has been optimized by different median filtering schemes. The final transmission matrix is shown in step V in Fig. S4b.

**Supplementary Note 4│Performance summary of state-of-art on-chip spectrometers.**

| Structure | Footprint  (μm^2^) | Res (nm) | Footprint * Res  (mm^2^·nm) | BW  (nm) | BW/Res | CFR  (ch·mm^-2^) | Platform | Ref |
| --- | --- | --- | --- | --- | --- | --- | --- | --- |
| AWG | 1000000 | 1.6 | 1.6 | 10 | 6 | 6 | SOI | [21] |
| Disordered photonic structure | 100 × 50 | 0.75 | 0.004 | 25 | 33 | 6600 | SOI | [43] |
| Digital planar holography | 2000 × 2000 | 0.15 | 0.6 | 148 | 987 | 246 | Si_3_N_4_ | [20] |
| Spiral waveguide | 500 × 500 | 0.01 | 0.003 | 0.4 | 40 | 160 | SOI | [44] |
| MZI+MRR | 520 × 220 | 0.02 | 0.002 | 12 | 600 | 5244 | Si3N4 | [46] |
| Multimode waveguide | 500 × 200 | 0.16 | 0.016 | 5.8 | 36 | 360 | SOI | [51] |
| Disorder medium | 200 × 100 | 0.4 | 0.008 | 15 | 37.5 | 1875 | Si3N4 | [50] |
| Echelle grating | 3200 × 3200 | 2.5 | 25.6 | 200 | 80 | 7.8 | Si_3_N_4_ | [22] |
| Fourier Transform | 2400 × 500 | 0.2 | 0.24 | 20 | 100 | 83.3 | SOI | [38] |
| MRR + gratings | 310 × 215 | 0.2 | 0.013 | 60 | 300 | 4501.1 | SOI | [14] |
| Photonic Crystal slab | 210 × 210 | 1 | 0.044 | 200 | 200 | 4535.1 | SOS | [49] |
| Metasurfaces | 7000 × 7000 | 1.2 | 58.8 | 100 | 83.3 | 1.7 | Glass | [24] |
| MRR+AWG | 1150 × 1250 | 0.75 | 1.078 | 57.5 | 76.66 | 71.13 | Si_3_N_4_ | [23] |
| MRR+AWG | 270 × 200 | 0.2 | 0.011 | 70 | 350 | 6,481 | SOI | [28] |
| Disordered  micro-ring lattice | 1000 × 1000 | 0.015 | 0.015 | >40 | 2666 | 2666 | SOI | [18] |
| Engineered MZI | 1900 × 3700 | 0.01 | 0.07 | 200 | 20000 | 2845 | Si_3_N_4_ | [17] |
| Stratified waveguide filters | 4 × 10^6^ | 0.12 | 0.48 | 120 | 1000 | 250 | Si_3_N_4_ | [52] |
| MRRs + MZIs | 2000 × 7600 | 0.02 | 0.304 | 115 | 5750 | 378.3 | Si_3_N_4_ | [16] |
| Tunable Fourier Transform | 5500 × 6000 | 0.125 | 4.125 | 200 | 1600 | 48.5 | SOI | [42] |
| This work | 150 × 950 | 0.07 | 0.001 | 100 | 1428 | 10021 | SOI | This work |

BW: bandwidth. Res: resolution. CFR: channel-to-footprint ratio. The reference number is consistent with the manuscript.
